# Improving ozone-based processes for the degradation of paraquat and diquat as persistent pesticides

**DOI:** 10.1007/s11356-026-37798-6

**Published:** 2026-05-12

**Authors:** Joseph Soto-Verjel, Paula Serrano-Tarí, Salvador Villamizar, Ana Ruiz-Delgado, Aymer Maturana-Córdoba, Isabel Oller

**Affiliations:** 1 Department of Civil and Environmental Engineering, Instituto de Estudios Hidráulicos y Ambientales IDEHA, Universidad Del Norte, Barranquilla, Colombia; 2 Department of Environmental Sciences, Grupo de Investigación en Procesos Ambientales GIPROAM, Universidad Francisco de Paula Santander, Cúcuta, Colombia; 3 CIEMAT, Departamento de Energía, Centro de Investigaciones Energeticas Medioambientales y Tecnologicas, Plataforma Solar de Almería Tabernas, Almería, Spain

**Keywords:** Diquat, Fe_3_O_4_, Photocatalytic ozonation, Paraquat, TiO_2_, Toxicity

## Abstract

The widespread use of bipyridylium herbicides like paraquat (PQ) and diquat (DQ) poses a significant environmental threat due to their high toxicity and recalcitrance. This study evaluates advanced oxidation processes based on ozonation as a robust alternative for their degradation. Simple ozonation was tested at various dosages (0.9 to 1.8 gh⁻^1^) to determine the oxidant’s efficiency in breaking down the parent molecules, while catalytic (Fe_3_O_4_) and photocatalytic (TiO_2_/solar) systems were evaluated to enhance mineralization and overcome ozone’s selectivity toward oxidation intermediates. Experiments progressed from 1 L lab-scale reactors to a solar compound parabolic collector (CPC) pilot system to assess scalability under complex conditions. The results showed that while simple ozonation effectively removed the parent herbicides (up to 83% for DQ), it was insufficient for complete mineralization. The addition of Fe_3_O_4_ did not significantly improve the process; however, photocatalytic ozonation at a pilot scale achieved the highest mineralization rates and total contaminant removal. Ecotoxicity assays using *Aliivibrio fischeri* revealed toxicity in generated transformation products, particularly at lower ozone dosages, necessitating optimized exposure times to ensure safe effluent discharge. Furthermore, by leveraging renewable solar radiation and optimizing the ozone dosage, the overall energy demand is significantly reduced, offering a highly sustainable and cost-effective approach. This work demonstrates the effectiveness of integrated solar ozonation processes for the treatment of point-source herbicide contamination.

**Graphical Abstract:**

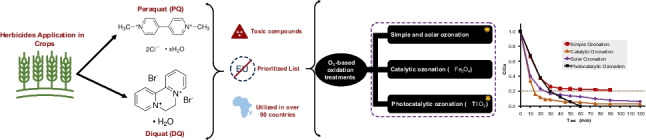

## Introduction

The exponential growth of the global population has made agrochemicals an important tool for intensifying agricultural activity and increasing crop yields (Valbuena et al. 2021). However, the excessive use of these chemicals can leave residues with negative effects on human health and the environment (Zhang and Yang 2021). Herbicides represent the most widely used category of agrochemicals in the global agricultural sector (FAO, 2025), utilized for the management and eradication of weeds (Rosic et al. 2020). Bipyridylium quaternary ammonium compounds form a group of non-selective contact herbicides commonly referred to as “quats,” represented by mepiquat, chlormequat, difenzoquat, paraquat (PQ), and diquat (DQ), with the latter two being the subject of extensive research (Belmonte et al. 2022). These agrochemicals are commonly found in surface water and soil matrices (reaching concentrations up to 1.5 mg/L for PQ) (Mendoza-Marín et al. 2010), alongside wash waters from agricultural spraying equipment (up to 930 mg/L for herbicides like 2,4-D) (Alza Camacho 2018), highlighting the magnitude of the environmental contamination problem***.*** This increased attention can be attributed to their notable properties, which include high water solubility (620 and 100 mgmL^−1^, respectively) (NHI, 2025a, 2025b), low volatility, and slow biodegradation (Liu et al. 2022).

Nevertheless, PQ exhibits one of the highest acute toxicities among herbicides (Tsai 2013), being 28 times more toxic than glyphosate (Walsh and Kingwell 2021), which justifies its ban in several countries (Jara et al. 2024). Due to this severe toxicity, these herbicides cannot be left to undergo slow, natural degradation in the environment; therefore, treating these effluents at the emission source (agro-industrial wastewater) using pilot-scale advanced oxidation processes represents a critical approach to destroy the active molecules and reduce their ecotoxicity prior to discharge, directly solving the environmental persistence issue*.* As a result, both PQ and DQ are included in the European Union (EU) prioritized list of herbicides of potential concern, with a specific ban on the application of PQ (Belmonte et al. 2022). However, PQ is still used in over 90 countries, including Colombia, Mexico, and Brazil. This has significant implications for South America, given its status as a fundamental agricultural region globally.

Current literature reports various treatment processes for the removal and/or degradation of these contaminants, highlighting three main categories: heterogeneous and homogeneous photocatalysis, biological processes, and adsorption. Regarding heterogeneous photocatalysis, the use of the TiO_2_ Degussa P25 catalyst and its modification as Ti_1_-xFexO_2_ (*x* = 4%) in lab-scale reactors stand out for the degradation of PQ and DQ in deionized water matrices (Florêncio et al. 2004). Under varied conditions of pH (3–8) and temperature (20–40 °C), no degradation occurred in the absence of UV light or the catalyst, demonstrating that the simultaneous presence of both factors is strictly necessary for the degradation of these herbicides. Other studies have evaluated UV and solar light along with TiO_2_-doped catalysts, achieving degradation rates of up to 98% (Kaur et al. 2019b), as well as alternative catalysts like ZnO·WO_3_ (Tariq et al. 2022) or photo-Fenton processes using industrial waste (Pandey et al. 2023). Considering biological processes, biodegradation faces significant kinetic limitations when dealing with highly recalcitrant molecules, requiring long acclimation periods and showing restricted efficiency without the intervention of additional chemical treatments (Jindakaraked et al. 2021)*.* Several successful studies have also been conducted on adsorption treatments using materials such as magnesium silicate fibers (Li et al. 2017), nanoporous carbon (Chanpee et al. 2022), zeolite (Sirival et al. 2018), and activated carbon (Jafari et al. 2022).

Despite the reported advances, previous studies present limitations that hinder their practical application for the efficient removal of bipyridylium herbicides in real aqueous matrices. In the case of photocatalytic processes, although significant degradation is achieved, they require prolonged residence times (Amiri et al. 2021) and have mostly been evaluated at low initial concentrations in distilled water. Biological processes show reduced performance against these contaminants (Pathak et al. 2025), and adsorption methods only physically separate the pollutant, entailing the subsequent management of the saturated material (Dotto and McKay 2020). By shifting the focus toward pilot-scale advanced oxidation processes under more complex matrices and strongly oxidizing conditions, the present study intends to overcome these barriers, representing a necessary step toward a real and scalable environmental solution. In this context, ozonation must be considered a robust strategy capable of offering faster reaction kinetics and the complete mineralization of bipyridylium herbicides like PQ and DQ (Aidoo et al. 2023; Solís et al. 2019). Ozonation-based advanced oxidation processes (AOPs) have been proven to be effective in removing these contaminants (Zarei et al. 2023). However, although ozone is highly effective against the parent herbicides (PQ and DQ), its strong selectivity provokes slow reactions; therefore, simple ozonation is limited in achieving complete mineralization (Jin et al. 2023). By combining ozone with catalysts and/or in the presence of solar or UV light, reaction kinetics were enhanced, and higher mineralization was achieved.

Regarding heterogeneous catalytic and photocatalytic ozonation (Malik et al. 2020; Malvestiti et al. 2019), various investigations have demonstrated the application of metal-oxide nanoparticles. Fe-based catalysts are widely used due to their abundance, non-toxicity, and magnetic properties, as in the case of Fe_3_O_4_ (Rekhate and Srivastava 2020). Metal-catalyzed ozonation improves the removal efficiency of organic compounds produced as intermediates (Li et al. 2013), which is supported by multiple studies using Fe_3_O_4_ for diverse contaminants (Castro et al. 2019; Chávez et al. 2019; Chávez et al. 2020; Prada-Vásquez et al. 2021; J. Wang and Bai 2017).

The main objective of this research is to evaluate and compare the efficiency of direct, catalytic, and photocatalytic ozonation at a pilot-plant scale for the removal of PQ and DQ. Furthermore, this study aims to assess the ecotoxicological outcomes of the generated intermediates to determine the most sustainable and safe treatment alternative for field application*.* Simple ozonation (SO), catalytic ozonation (CO), and photocatalytic ozonation (PCO) were carried out at a pilot-plant scale, alongside preliminary lab-scale experiments. The catalysts used were magnetite (Fe_3_O_4_) and TiO_2_ Degussa P25; acute toxicity tests were included to optimize their application for water reuse.

## Materials and methods

### Reagents and water matrix

PQ and DQ with a high-purity grade (≥ 98% and ≥ 95%, respectively) were purchased from Sigma–Aldrich. DQ Reglone® technical product was bought from Syngenta (CAS number 85–00–7), containing 200 gL^−1^ of active ingredient DQ (dibromide) (see Table 1). Commercial DQ product was acquired from Syngenta S.L, though analytical standard PQ was evaluated and used for comparison as the technical product is prohibited and not commercially available in Europe.

**Table 1:**
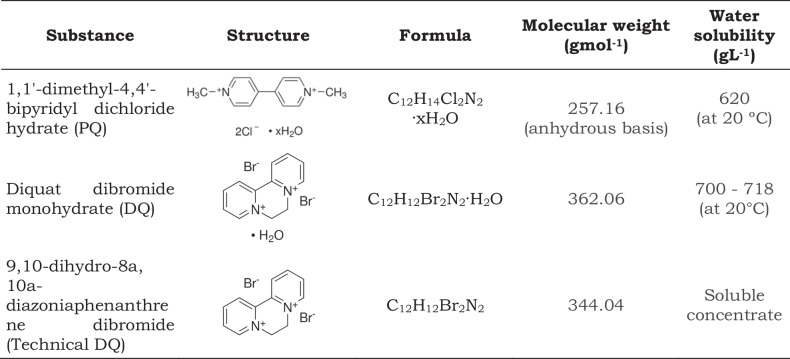
Physicochemical properties and structure of the target contaminant

Iron (II, III) oxide nano-powder (50–100 nm particle size (SEM) and 97% trace metal base), CAS number 1317–61-9> (Magnetite), molecular weight 231.53 gmol^−1^, density 4.8–5.1 gmL^−1^ at 25 °C and color black, obtained from Sigma–Aldrich, was tested. In addition, commercial Evonik P25-TiO_2_ (21 nm primary particle size, by TEM, density 140 gL^−1^, surface area of 50 m^2^g^−1^ and white color), a semiconductor, was studied as a reference semiconductor photocatalyst.

Demineralized water (DW) was obtained from the reverse-osmosis + electrodeionization plant located at Plataforma Solar de Almería (Spain) (conductivity < 10 µS/cm and inorganic and dissolved organic carbon below 0.5 mg L-1). Panreac supplied acetonitrile (ACN) and Honeywell|FlukaTM supplied ammonium acetate, which were used as mobile phases for analyzing PQ and DQ by liquid chromatography. Hydrochloric acid used for pH adjustment was bought from J.T. Baker. Potassium Indigo trisulfonate (C_16_H_7_K_3_N_2_O_11_S_3_) was obtained from Sigma–Aldrich; Ortho-Phosphoric Acid (85%) was purchased from Merck, and Sodium Bisphosphate (NaH_2_PO_4_) was bought from J.T. Baker. These were used for the indigo method to measure the dissolved ozone in samples. Ultrapure water (Milli-Q) was used for the PQ and DQ stock solutions, as well as for the indigo-method stock solution.

### Experimental set-up and procedure

#### Lab scale experiments

Preliminary experiments performed at the laboratory scale included: (i) solar photolysis of PQ and DQ, (ii) iron leaching from magnetite (Fe_3_O_4_), as the selected catalyst, under acidic-pH (3.0) and natural-pH (5.5) conditions, (iii) adsorption of analytical PQ on magnetite at natural pH, and (iv) UV photocatalysis at two concentrations of magnetite in both acidic and natural-pH conditions.

**Fig. 1:**
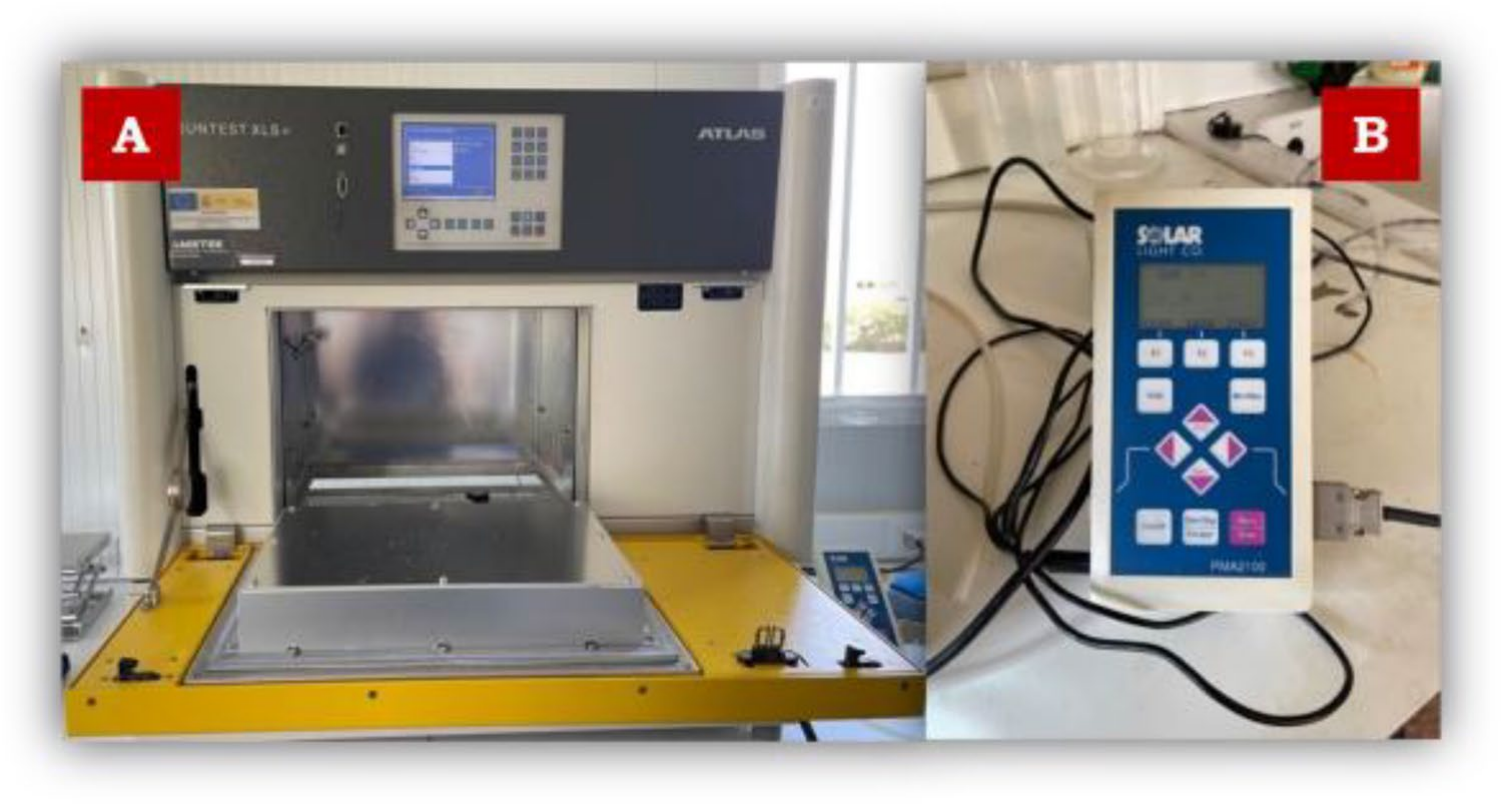
**A** Solar simulator system: SUNTEST XLS + (Atlas). **B** Portable UVA radiometer (Solar Light Co., Inc)

All lab-scale assays were performed in a 1 L borosilicate-glass reaction vessel under continuous magnetic stirring at 350 rpm. The experiments under simulated solar radiation were performed inside a solar simulator chamber (Atlas-SunTest XLS +), programmed for total radiation of 365 Wm^−2^ (300–800 nm) and UVA-UVB radiation of 30 Wm^−2^ (300–400 nm). Solar Light Co., Inc (Philadelphia), model PMA2111 was used as a UVA detector (see Fig. 1). These experiments were done in DW spiked with 10 mgL^−1^ of PQ or DQ and 1 gL^−1^ or 3 gL^−1^ of magnetite at pH 3 (adjusting with hydrochloric acid) and natural pH (5.5). After the addition of the iron nanoparticles, a mixing period of 5 min was allowed, and the solar simulator was immediately turned on. Samples were taken at predetermined and regular time intervals to analyze the PQ and DQ concentrations by HPLC–DAD. The volume extracted per sample was 7 mL, which represented less than 5% of the total initial volume.

Selection of Fe_3_O_4_ doses (1.0 and 3.0 gL^−1^) was based on their proven performance in AOPs. A concentration of 1.0 gL^−1^ is ideal for ensuring high efficiency with minimal iron leaching (He et al. 2015; Huaccallo-Aguilar et al. 2021). Conversely, the 3.0 gL^−1^ dose was chosen to enhance degradation without triggering the adverse effects of particle agglomeration and light scattering typical of higher loadings (Liu et al. 2025). Moreover, this latter value represents the optimal dose reported for catalytic ozonation applied to complex matrices (Becerra-Moreno et al. 2025).

#### Ozonation treatment

**Fig. 2:**
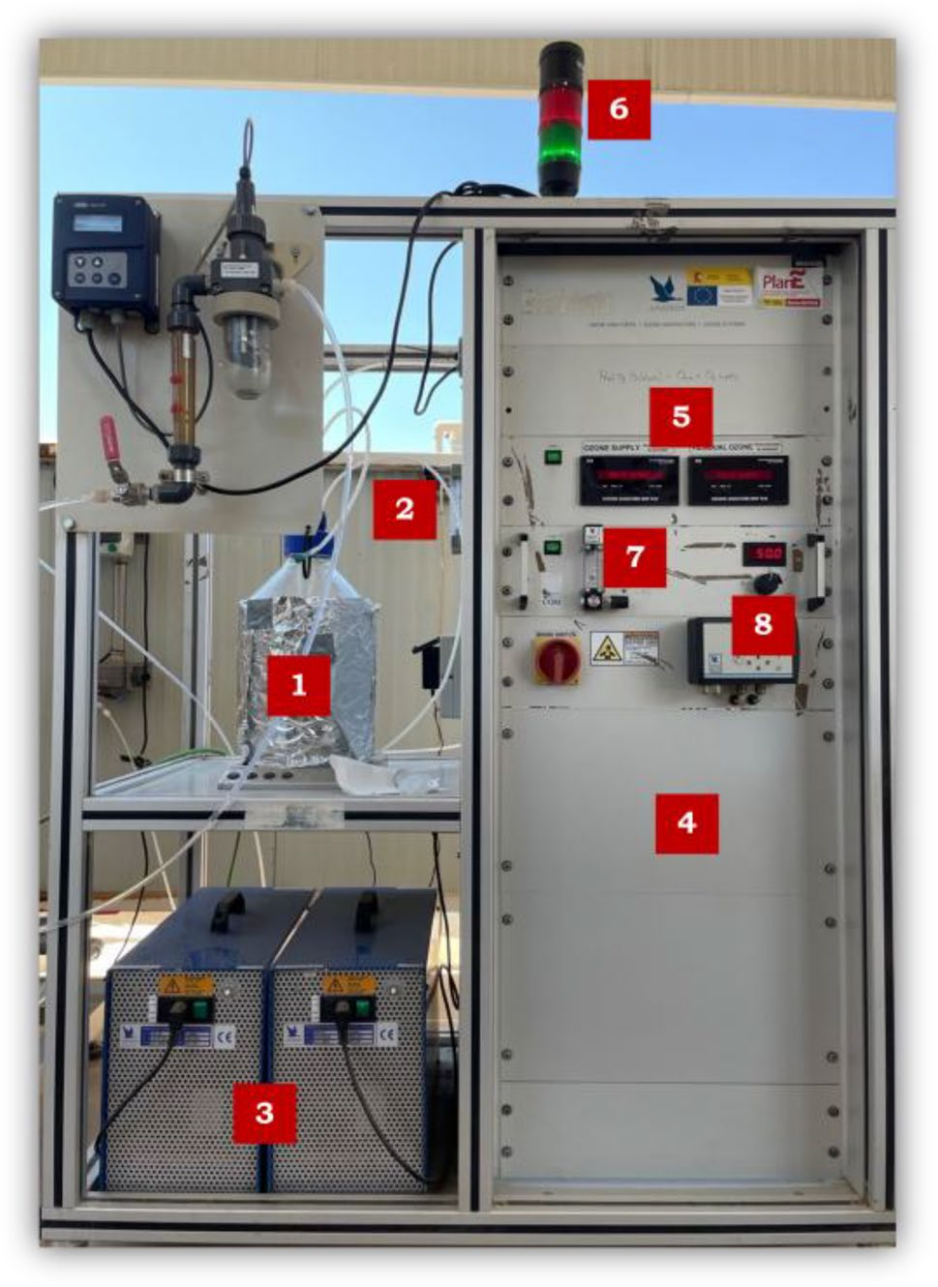
Ozonation pilot plant (ANSEROS SL). Glass reactor (1); flowmeters (2); oxygen generators (3); ozone generator (4); ozone analyzers (5); ambient ozone detector (6); ozone generation flow rate (7); percentage ozone generation (8)

**Fig. 3:**
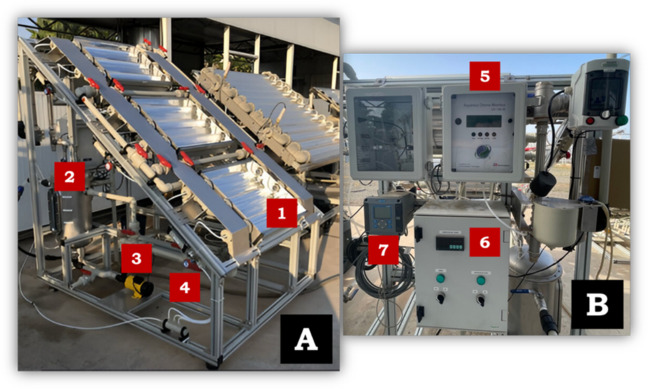
CPC pilot plant combined with ozonation system. **A** Solar photocatalytic reactor with ozone diffusers. **B** Electrical panel CPC module (1); lung tank (2); recirculation pump (3); manifold (ozone input to CPC) (4); dissolved ozone analyzer (5); control of pump start-up and tank temperature (6); pH and temperature control (7)

A single system was used for the generation of ozone gas, which consists of a flow meter, two oxygen generators (ANSEROS SEP 100), an ozone generator, and two non-dispersive UV gas-phase analyzers (Ozomat GM-6000-OEM) (see Fig. 2). It is coupled to a thermal (300 °C) and catalytic ozone destructor to prevent the release of ozone into the environment. An ambient ozone detector (ANSEROS, SEN 6060-S) is connected to an alarm for the continuous monitoring of ambient ozone concentrations and to prevent risks to the operator. Operating under two independent configurations, this single generation system was either coupled to the glass reactor for single and catalytic ozonation (see Fig. 2) or connected to the CPC solar reactor for photocatalytic processes (see Fig. 3). Ozonation and catalytic ozonation processes were carried out in a 10 L borosilicate glass reactor. The reactor was equipped with a microbubble diffuser for O_3_ injection and a continuous stirring system to ensure a homogeneous mixture (see Fig. 2). For PQ, DQ, and DOC quantification, representative samples were collected via syringe from the middle of the water column.

Photocatalytic ozonation experiments were performed at pilot-plant scale in a solar compound parabolic collector (CPC) installed in Plataforma Solar de Almería (latitude 37° N, longitude 2.4° W) (see Fig. 3). The pilot plant has three modules of 0.28 m^2^ of collector surface. Each one is composed of three borosilicate tubes of 50 mm external diameter, 2.5 mm thick, and 700 mm long. The three collector modules are configured to operate in series; however, the system can run using one, two, or all three modules. In this work, only one collector module was used, corresponding to an operating volume of 12 L and an irradiated volume of 2.4 L. An aqueous ozone monitor (UV-106-W model) was located at the outlet of the solar collector modules, and a Pt-100 temperature probe was positioned in the lower part of the buffer tank. For the measurements of compounds (PQ or DQ) and COD, samples were collected directly from the sampling valve located at the outlet of the CPC module.

In a typical run, an air stream was preliminary recirculated in the pilot plant system (borosilicate glass reactor or CPC) for a minimum period of 1 h to dry the whole system. Subsequently, the ozone destructor was turned on and PQ or DQ solution (10 mgL^−1^) at natural pH was added to the glass reactor. Next, the oxygen generator was turned on and the flow rates (inlet and outlet) were stabilized for 20 min at 0 g/Nm^3^. The ozone generation flow rate was set to 60 Lh^−1^ and the generation percentage at 50, 25, or 10% depending on the experiment. At this point, the ozone generator was turned on, and the experiment began. For the CPC reactor, before starting the ozonation, the solution was recirculated for 5 min in the system, after this time the ozone generator was turned on, and the collector was uncovered; in cases where the experiment was run without sunlight, the collector was kept covered. Samples were taken at specific time intervals. Immediately after sampling, a small flow of inert gas (N_2_) was bubbled to remove residual ozone and stop the reaction for analytical measurements.

Solar UV-A irradiance (Wm^−2^, 280–400 nm) was monitored using a pyranometer (Kipp&Zonen, CUV5, Netherlands) tilted at 37° as the CPC reactor. Solar irradiation has been quantified according to Eq. (1) (Berruti et al. 2023).

1 $${Q}_{UV,n}={Q}_{UV,n-1}+\frac{{UV}_{n}.{A}_{i}.\Delta t}{{V}_{t}.1000}$$

where ${Q}_{UV,n}$ is the accumulated UV energy per unit of volume (kJL^−1^) at time $n$; ${UV}_{n}$ is the average solar ultraviolet incident radiation on the irradiated area (Wm^−2^); $\Delta t$ is the experimental time between samples ($\Delta t = {t}_{n} - {t}_{n-1}$); $Ai$ is the illuminated area (m^2^) and $Vt$ is the total volume of treated water (L).

### Analytical determinations

Quantification of PQ and DQ was performed by high-performance liquid chromatography (HPLC) (Agilent Technologies, Series 1100) with a UV-DAD detector. A reverse-phase C18 (2) Luna analytical column (150 × 3 mm, 100 Å, 5 μm) (Phenomenex, Torrance, CA, USA) was used with 0.5 mLmin^−1^ flow rate with the mobile phase of 30% of ACN and 70% of ammonium acetate (20 mM) at pH 3. The column was kept at 30 °C and the total analysis running time was 5 min. PQ was obtained by UV-DAD at 254 nm (Yao et al. 2021) and DQ at 310 nm (Melo et al. 2009). The injection volume was 20 μL. All samples were prepared by filtering 7 mL of the sample through a 0.22 μm PTFE Millipore syringe filter and washing the filter with 3 mL of ACN mixed with the filtered sample to remove any compound adsorbed on the filter and to preserve the sample. The limit of detection (LOD) was 0.5 mg L^−1^ and the limit of quantification (LOQ) was 1.0 mg L^−1^ for both pollutants.

Dissolved organic carbon (DOC) was measured with a Shimadzu TOC-VCN analyzer. These samples were filtered through 0.45 μm Nylon Filter-lab filters prior to injection. Iron concentration was quantified following ISO 6332 using a Unicam UV/Vis UV2 spectrophotometer (at 510 nm). Ozone concentration in solution was measured by the indigo method based on the decolorization of indigo trisulfonate (600 nm) by ozone (American Public Health Association (APHA) et al., 2023; Bader and Hoigné, 1981).

### Acute toxicity analysis

#### *A. fischeri* bioluminescence inhibition test

Ecotoxicity assessment was performed with the psychrophilic marine bacterium, *Aliivibrio fischeri* (NRRL B-11177), with the commercial BioFix Lumi-10 kit. The inhibitory effect of water samples on the bioluminescence emission of *A. fischeri* was assessed following the standard procedure (ISO-11348-3:2007) (International Organization for Standardization (ISO), 2007). Bioluminescence emission was measured using a BioFix® Lumi-10 luminometer (Macherey-Nagel GmbH & Co. KG, Duren, Germany) after 15 and 30 min of sample exposure. Toxicity results were expressed as the bioluminescence inhibition percentage (BI %) compared to an uninhibited control. A BI < 50% indicates that the water sample does not pose a harmful effect on the receiving aquatic environment.

#### *A. franciscana* acute toxicity analysis

Acute toxicity was assessed using the commercial bioassay Artoxkit M (Microbio Test Inc., Belgium), following the standardized protocol “*Artemia* Toxicity Screening Test for Estuarine and Marine Waters” (Microbio Test Inc. n.d.). The test employs larvae of the brine shrimp *Artemia franciscana*, obtained by hatching dormant cysts in synthetic seawater under controlled conditions of artificial light and at 25 °C. Synthetic seawater with a salinity of 35% was used both as the incubation medium for cyst hatching and as the diluent for preparing the series of pollutant concentrations. A negative control (standard culture medium without herbicides or effluent) was used to ensure baseline viability and the natural survival of the test organisms against the water matrix. Simultaneously, a positive control (using a known reference toxicant such as K_2_Cr_2_O_7_) was implemented to verify that the organism batch presented adequate sensitivity and responded predictably to toxicity, thereby guaranteeing the validity of the results compared to the oxidized samples.

After 24 h of exposure, the number of surviving organisms was determined using a dissecting microscope, and the percentage of mortality was subsequently calculated (Ponce-Robles et al. 2019).

## Results and discussion

### Photocatalytic degradation experiments at lab-scale

Solar photolysis results of 10 mgL^−1^ analytical PQ and DQ demonstrated their non-degradation after 6 h of exposure (Liu et al. 2022), highlighting their slow degradation kinetics in this process (Marien et al. 2019). Combining solar photolysis with other processes, such as the use of catalysts or the application of advanced oxidation techniques, could significantly enhance the degradation and mineralization of these persistent compounds.

In this context, the assessment of metallic catalysts, such as magnetite ($Fe_3O_4$), offers a promising alternative to enhance the generation of reactive oxygen species and is fully compatible with the activation with solar radiation (Niveditha and Gandhimathi 2020). The results showed that iron leaching significantly increased under acidic pH and solar-UV light exposure. Furthermore, comparing the outcomes from solar photocatalysis with $3 {\mathrm{gL}}^{-1}$ $of Fe_3O_4$, it was observed that increasing the catalyst concentration also increases the residual iron content. For a $Fe_3O_4$ dosage of$1 {\mathrm{gL}}^{-1}$, dissolved iron concentrations rapidly decreased when exposed to solar radiation at natural pH (0.06 mgL^−1^), whereas, in the dark, higher leaching is observed under acidic pH conditions (0.29 mgL^−1^). For the $3 {gL}^{-1}$ dosage, iron leaching is more pronounced under solar radiation and acidic pH, reaching a final dissolved iron concentration of $2.6 {mgL}^{-1}$, compared to $0.8 {mgL}^{-1}$ for the $1 {\mathrm{gL}}^{-1}$ dosage under the same operating conditions.

This analysis underscores the importance of iron leaching as a potentially beneficial mechanism in homogeneous photocatalytic processes. The released iron ions can act as secondary catalysts promoting hydroxyl radical formation and enhancing contaminant degradation (Ngulube et al. 2024).

Solar photocatalysis was performed with two concentrations of $Fe_3O_4$ (1 and $3 {gL}^{-1}$) under acidic (pH 3.3) and natural (pH 5.5) pH conditions, using a solution containing $10 {mgL}^{-1}$ of analytical PQ. There was no degradation of the contaminant under any condition studied during 300 min of treatment. Thus, the findings revealed that magnetite does not effectively function in catalytic or solar photocatalytic processes for the degradation and/or mineralization of PQ, and neither acts adsorbing the contaminant (Kaur et al. 2019a). These results together with the drawback of the high weight of this catalyst which difficult its proper mixing within a solar photoreactor, requiring high energy consumption, rules out its interest for the degradation of high concentrations of persistent pollutants.

In consequence, the effective degradation of PQ in aqueous solutions via solar photocatalysis requires the incorporation of modified catalysts (Moradi et al. 2023), the addition of oxidants (Desipio et al. 2018), and/or complementary strategies to achieve efficient degradation and mineralization.

### Ozonation treatment

Ozonation treatments were tested for the degradation of $10 {mgL}^{-1}$ of analytical PQ and both analytical and technical DQ. An 80% degradation threshold was established as target, considering the Swiss Federal Office of the Environment (FOEN) and Federal Water Protection Law (l′OEaux). Mineralization, measured as total organic carbon (TOC), was assessed at the end of each experimental condition.

To ensure that herbicide removal is solely attributed to the evaluated advanced oxidation processes, the effect of adsorption was analyzed. In the case of magnetite (Fe_3_O_4_), it was confirmed that PQ did not adsorb onto the catalyst surface; after 360 min in the dark (natural pH of 5.5, 1 gL^−1^ Fe_3_O_4_, constant stirring), no removal was observed. Similarly, photocatalytic assays with varying magnetite concentrations at both acidic and natural pH (see Section “Photocatalytic degradation experiments at lab-scale”) showed no evidence of removal or mineralization processes. Although adsorption onto titanium dioxide (TiO_2_) was not experimentally quantified in this study, the literature indicates that the affinity of cationic herbicides (PQ and DQ) for pure TiO_2_ is exceedingly low (Brigante and Schulz 2011). This adsorptive capacity increases significantly only when the material is supported, doped, or coated with negatively charged phases (M’Bra et al. 2019), which promotes selective adsorption prior to irradiation (Nghia et al. 2018).

#### Simple ozonation

**Fig. 4:**
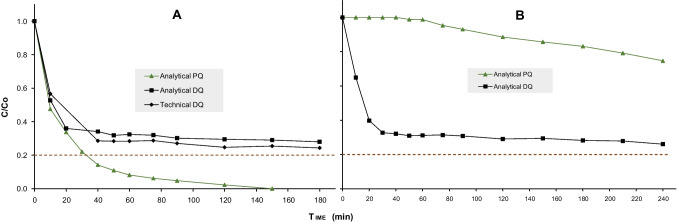
**A** Ozonation of analytical and technical DQ and PQ at 10 mgL^−1^ each but separately. **B** Ozonation of the mixture of both contaminants. Maximum O_3_ *dosage of 3 gh*^*−1*^. C/Co threshold = 0.2 (80% degradation)

As a preliminary test, Fig. 4A displays the results of ozonation applied to the oxidation of DQ and PQ in their different chemical forms and separately, using the maximum O_3_ dose ($$3 {gh}^{-1})$$, available in the pilot plant at Plataforma Solar de Almería facilities, to check the global performance of the system. Additionally, the treatment of a mixture of both compounds in the same water matrix is shown in Fig. 4B.

Eighty percent degradation of analytical PQ was achieved rapidly within 30 min of ozonation, with a degradation higher than 99% after 150 min. Final mineralization reached 71%, indicating effective removal of organic carbon, not only the removal of the parent contaminant. In contrast, analytical DQ exhibited slower degradation, failing to get the 80% threshold within 240 min of ozonation, but achieving a reduction of 74%. Final mineralization was 37%, highlighting the considerable resistance of DQ to ozonation; technical DQ showed similar behavior, which suggests the absence of a high amount of excipients or additives in the technical formulation normally included to improve the product solubility. These results also reveal the higher resistance and chemical stability of DQ compared to PQ mainly provoked by the difference in their structures due to the presence of a third ring (Teutli-Sequeira et al. 2020).

The results presented in Fig. 4B indicated that when analytical PQ and DQ were treated together in a mixture, the ozonation process exhibited greater selectivity toward DQ oxidation, achieving 74% degradation, while PQ achieved only 34% removal. Mineralization was limited to 26%, suggesting that the interaction between both compounds generates more resistant byproducts that are not easily degraded during ozonation (Khodkar et al. 2019), limiting the overall treatment efficiency.

The greater ozone selectivity observed toward DQ degradation in the mixture with PQ can be attributed to two complementary factors. First, the formulation of DQ as a dibromide introduces Br⁻ ions into the matrix, which readily react with ozone to generate reactive brominated species that contribute to indirect oxidation pathways and consequently enhance DQ transformation, maintaining the same behavior as when it was treated alone (Morrison et al. 2023; Naumov and von Sonntag 2008). Second, intrinsic differences in molecular structure and redox chemistry play a decisive role: while PQ and DQ share the bipyridyl core, the presence of an ethylene bridge (-CH_2_-CH_2_-) in DQ, absent in PQ, represents an aliphatic site that is more vulnerable and kinetically favorable to oxidative attack, particularly by hydroxyl radicals generated during the process (Hoigné and Bader 1976). In fact, comparative studies of AOPs have confirmed that the initial oxidative attack on DQ proceeds with higher reaction rate constants relative to PQ, resulting in faster degradation kinetics (Teutli-Sequeira et al. 2020).

**Fig. 5:**
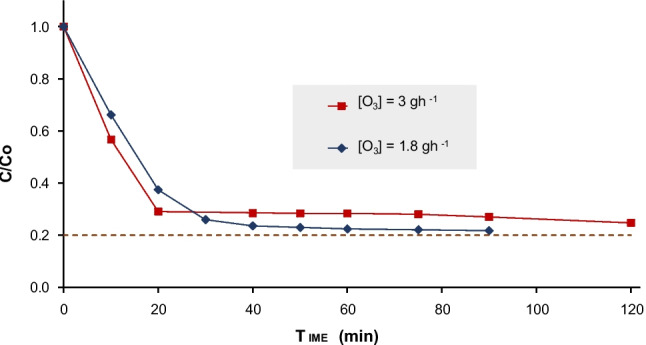
Ozonation of 10 mgL^−1^ of technical DQ at two extreme ozone doses

Given the extended use of technical grade PQ and DQ products from the commercial point of view, this study has also evaluated the ozonation efficiency on technical DQ, specifically Reglone®, a Syngenta formulation (described in Table 1, Section “Reagents and water matrix”). This selection has been done considering the greater resistance shown by DQ compared to PQ, enabling the optimization of oxidation processes. Figure 5 illustrates the results of ozonation applied to technical DQ using two different ozone doses ($1.8$ and $3 {gh}^{-1}$).

For the low ozone dosage ($1.8 {gh}^{-1}$), after 90 min of treatment, degradation reached 78% with a mineralization of 39%. However, most of the degradation (74%) occurred within the first 30 min. Finally, for the high ozone dosage ($3.0 {gh}^{-1}$), 80% degradation was achieved over the total treatment time (120 min), with most of the degradation (73%) occurring in the first 20 min and a final mineralization of 47%.

The results indicated that increasing the ozone dose did not significantely improved the removal efficiency of DQ, or the mineralization rate (only 8%). In consequence, a higher consumption of resources and energy for applying a higher dosage of ozone is not justified in this case. In addition, the main interest on applying a strong and potentially expensive oxidation step, such as ozonation, would be degrading the parent recalcitrant contaminants, such as DQ and PQ, and so generating more biodegradable byproducts. Therefore, a dosage of $1.8 {gh}^{-1}$ was finally selected, offering a balanced trade-off between contaminant degradation and mineralization (Fu et al. 2018). It has been previously reported that higher doses of ozone are required to degrade intermediate byproducts and achieve higher levels of mineralization (Cruz-Alcalde et al. 2017), consistent with the behaviour observed in this study. However, ozonation can produce oxidation byproducts with potential toxicity, even higher that the parent compounds, and even with higher probability when lower doses of ozone are applied and less mineralization achieved. This highlights the importance of combining ozone with solar light and catalysts to potentially improve the mineralization rate, as this approach would increase the generation of hydroxyl radicals (Bourgin et al. 2017).

#### Catalytic ozonation

**Fig. 6:**
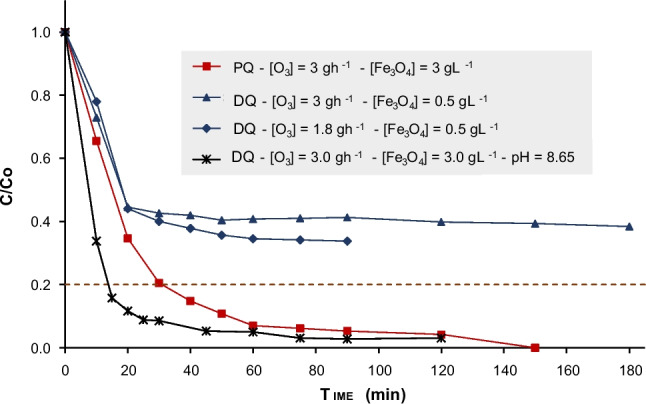
Catalytic ozonation of analytical PQ and technical DQ

Catalytic ozonation was tested for improving target contaminant degradation. Considering the similar behavior of analytical and technical DQ when applying only ozone, it was decided to continue evaluating the degradation performance on technical DQ, as it is the actual product frequently used and less costly than the analytical one. Figure 6 presents the results of catalytic ozonation (CO) applied to the oxidation of technical DQ and analytical PQ, where two ozone doses and two concentrations of magnetite as a catalyst were evaluated. In addition, it illustrates the results of an additional condition at basic pH (8.65) with the maximum dosage of ozone and catalyst. This approach to CO for the removal of specific contaminants and its relationship with variables such as catalyst dosage, ozone dosage, and pH is supported by previous studies (Kermani et al. 2018).

As analyzed from results shown in Fig. 5, an ozone dosage of $1.8 {gh}^{-1}$ achieved a maximum degradation of technical DQ of 78% with 39% of mineralization after 90 min of treatment. However, when introducing the catalyst at $0.5 {gL}^{-1}$ under similar operating conditions, degradation decreased to 66%, with a very low mineralization of 7%. The initial degradation efficiency remained high (55% in the first 20 min of treatment), but further improvement was minimal. When $3.0 {gh}^{-1}$ of ozone dosage was applied (Fig. 5), 79% degradation and 47% mineralization were observed after 120 min of treatment. However, with the addition of $0.5 {gL}^{-1}$ of catalyst, total degradation decreased to 63%, with a limited increase in the first 20 min (56%) and only a marginal improvement (8%) over the 120 min. The final mineralization reached 21%.

Therefore, introducing $0.5 {gL}^{-1}$ of magnetite into the ozonation system resulted in a reduction in the degradation and mineralization percentages of technical DQ compared to the application of ozone alone. In dark conditions, magnetite promotes the breakdown of ozone molecules into hydroxyl radicals (•OH) and oxygen species, substituting the ozone oxidation reaction with the target contaminant by the one carried out by •OH (Singh et al. 2024). The results shown in Fig. 6 reveal the lower efficacy of •OH to specifically degrade the molecule of DQ compared to the high efficacy shown by ozone (Jin et al. 2021; Xiong et al. 2019), and so highlight the limited contribution of the catalyst in this specific context (Fasce et al. 2023).

Technical DQ degradation was also tested at basic pH (8.65), higher ozone dosage ($3\;gh^{-1}$), and higher catalyst concentration ($3\;gL^{-1}$), with the main objective of increasing the production of •OH and so, improving degradation and mineralization percentages. 85% threshold of technical DQ degradation was exceeded within 15 min of treatment, and final mineralization of 22% was achieved. It is clear that increasing the oxidation capacity of the whole system, significantly improved the removal of the parent contaminant, but it is also evident that while increasing the catalyst concentration promoted a faster initial degradation of the parent compound, attaining high mineralization rates still remained challenging (Niu et al. 2024).

In the case of analytical PQ, when adding $3.0 {gL}^{-1}$ of magnetite, similar degradation levels were obtained as when only a dosage of $3 {gL}^{-1}$ of ozone was applied, reaching 80% degradation in the first 30 min with a total mineralization of 62%. Again, at a high ozone dosage ($3 {gh}^{-1}$) with the addition of the magnetite, mineralization slightly improved compared to only ozonation, as commented above.

CO results demonstrated that using magnetite was not beneficial in enhancing the degradation or mineralization of DQ and PQ under the evaluated conditions. For DQ, the catalyst may interfere with the formation and activity of ozone-derived radicals. For PQ, simple ozonation proved to be sufficiently effective. These findings suggest that catalytic ozonation with magnetite would be more effective when employed in combined processes, such as with light, specific pH adjustments, or doped catalysts (López et al. 2023).

#### Photocatalytic ozonation

**Fig. 7:**
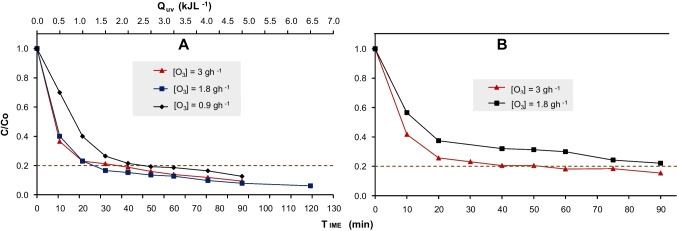
**A** Solar ozonation of technical DQ at three ozone doses. **B** Ozonation at two ozone doses in the CPC of technical DQ

The results of solar ozonation applied to technical DQ in the CPC reactor with the evaluation of three ozone doses are shown in Fig. 7A. Figure 7B shows the results under simple ozonation conditions (without solar radiation) in the same CPC reactor, aimed at highlighting significant differences in process efficiency.

The combination of an ozone dose of $3 {gh}^{-1}$ solar radiation allowed the degradation threshold to be reached in 35 min (5 min less), with an accumulated energy of $1.9 {kJL}^{-1}$ and achieving 17% of mineralization (at the end of the treatment, 90 min). Notably, a rapid degradation of 64% of the contaminant was achieved within the first 10 min. In contrast, the process without solar radiation reached the same degradation threshold in 50 min but showed no mineralization after 90 min of treatment (Fig. 7B).

For the intermediate ozone dose of $$1.8 {gh}^{-1}$$, in the first 10 min, 60% of the DQ was degraded, demonstrating a high initial process efficiency. However, without solar radiation, the degradation threshold was not reached after 90 min of treatment, achieving only 78% of degradation (Fig. 7B). Additionally, within the first 10 min, only 43% of the contaminant was degraded 7% less than with solar radiation. The solar radiation had more influence since the target degradation % was reached at 25 min (accumulated energy of $$1.5 {kJL}^{-1})$$ while in SO the same % was reached at 90 min of treatment.

Finally, at the lowest dose of $0.9 {gh}^{-1}$, the degradation threshold was reached in 50 min, with an accumulated energy of $1.9 {kJL}^{-1}$ and a mineralization of 6%. In this case, the initial degradation percentage in the first 10 min was lower (30%), approximately 30% less than with higher doses. In contrast, in simple ozonation experiments, both mineralization and the DQ degradation rate were significantly limited.

The results demonstrated that solar radiation has a substantial impact on the efficiency of the ozonation process, particularly in enhancing degradation rates and mineralization capacity (Linares-Hernández et al. 2022). Higher ozone doses ($3 {gh}^{-1}$) were more effective for rapid degradation but incurred higher energy consumption, whereas solar radiation enhanced mineralization even at intermediate or lower doses ($1.8 {gh}^{-1}$ and $0.9 {gh}^{-1}$). This highlighted that the ozone-sunlight combination not only accelerates contaminant removal but also contributes to the conversion of recalcitrant byproducts (Ribeiro et al. 2024). However, despite improving initial degradation efficiency, especially at lower doses, solar radiation reduces available ozone, limiting the generation of reactive species required for further mineralization (Shirkoohi et al. 2020).

**Fig. 8:**
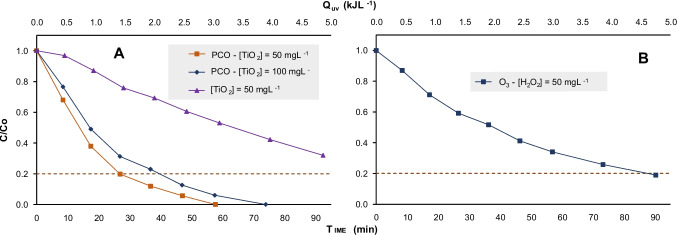
**A** Photocatalytic ozonation and photocatalytic process of technical DQ. **B** O_3_-H_2_O_2_ process of technical DQ. *Dosage O*_*3*_ = *1.8 gh*^*−1*^

Photocatalytic ozonation using TiO_2_ as the reference catalyst has been also evaluated at $50$ and $100 {mgL}^{-1}$, for the degradation of the technical DQ in the CPC reactor pilot plant and combined with an ozone dose of $1.8 {gh}^{-1}$. The results are shown in Fig. 8A. Additionally, the figure includes results from only photocatalysis with $50 {mgL}^{-1}$ of $TiO_2$. After 90 min of treatment and an accumulated energy of $4.9 {kJL}^{-1}$, the degradation threshold was not achieved, and mineralization reached only 7%. In the photocatalytic ozonation at a $TiO_2$ concentration of $100 {mgL}^{-1}$, the degradation threshold was reached in 42 min, with an accumulated energy of $2.0 {\mathrm{kJL}}^{-1}$, and 51% of the contaminant was removed in the first 20 min. Mineralization reached 56%, requiring a total accumulated energy of $4.8 {\mathrm{kJL}}^{-1}$. At the lower $\mathrm{TiO}_2$ concentration of $50 {\mathrm{mgL}}^{-1}$, the degradation threshold was achieved in 28 min, with an accumulated energy of $1.4 {\mathrm{kJL}}^{-1}$ and 62% of the contaminant was removed in the first 20 min. Mineralization was higher, 79%, requiring a total accumulated energy of $4.8 {\mathrm{kJL}}^{-1}$, in concordance with the well-known optimum TiO_2_ concentration in CPC of $50 {\mathrm{mgL}}^{-1}$ (Šuligoj et al. 2021). These results clearly show that the addition of ozone significantly enhances the process, consistent with previous studies highlighting ozone’s role in generating reactive species, such as hydroxyl radicals, in photo-assisted systems (Rodríguez et al. 2019).

In addition, and with the aim of comparing with another potentially interesting AOP based on the addition of H_2_O_2_ to ozonation, Fig. 8B illustrates the experiments carried out for the degradation of technical DQ with ozonation combined with $50 {mgL}^{-1}$ of $H_2O_2$.

In the peroxono experiment, the degradation threshold was achieved in 85 min requiring an accumulated energy of $4.8 {kJL}^{-1}$ and achieving a mineralization rate of 26%. While effective in terms of degradation, the results showed that the combination of $TiO_2$ and ozone achieved a faster initial degradation rate and greater mineralization. A similar trend was observed when comparing photoatalyitic ozonation to solar ozonation processes, emphasizing the conversion of byproducts into simpler and more biodegradable compounds (Kajitvichyanukul et al. 2022).

### Acute toxicity analysis

Acute toxicity evaluation during the degradation of technical DQ under various experimental conditions used an untreated control sample (10 mgL^−1^ DQ in DW) as a baseline reference. Additionally, negative control samples consisting of the water matrix subjected to the same oxidation conditions but without the initial addition of the herbicide were analyzed; the baseline behavior of these controls confirmed that any alteration in bioluminescence during the assays stems strictly from the target compound and its transformation products.

Three experimental conditions of only ozonation were selected, corresponding to ozone doses of 0.9, 1.8, and 3.0 gh^−1^. In the case of solar ozonation, two representative treatments were chosen: one with a low ozone dose (0.9 gh^−1^) and another with the maximum ozone dose applied (3.0 gh^−1^). All experiments were conducted under a total exposure time of 90 min.

One configuration for catalytic ozonation was selected: the highest magnetite concentration (3 gL^−1^), combined with the maximum ozone dose (3.0 gh^−1^) at pH 9, with a total reaction time of 240 min. Regarding photocatalytic ozonation, the experiment showing the best performance was considered, corresponding to a TiO_2_ concentration of 50 mgL^−1^ in combination with the intermediate ozone dose (1.8 gh^−1^) and a total exposure time of 90 min. Finally, O_3_/50 mgL^−1^ H_2_O_2_ treatment was also evaluated, with an ozone dose of 1.8 gh^−1^.

This selection of representative ozonation-based treatments provided a comparative framework to assess differences in potential bio-organisms inhibition, as well as the residual toxicity of the oxidation processes applied to the degradation of technical DQ.

#### *A. fischeri* bioluminescence inhibition test

The efficacy of ozonation in reducing the residual toxicity of technical DQ depended more on the type of process applied than on the simple removal of the contaminant. The results obtained with *Aliivibrio fischeri* revealed clear patterns when comparing the five strategies evaluated: simple ozonation, solar ozonation, catalytic ozonation, photocatalytic ozonation, and ozonation with H_2_O_2_, as summarized in Table 2.

**Table 2:** Toxicity results for *A. fischeri* for each treatment final sample

No	Sample	% Effect on bioluminescence	
		15 min	30 min
0	Inicial concentration technical DQ: 10 mgL^−1^	75.0 I ± 0.0	55.0 I ± 0.0
1	[O_3_]: 0.9 gh^−1^	83.0 S ± 0.0	58.0 S ± 1.4
2	[O_3_]: 1.8 gh^−1^	25.5 I ± 3.5	47.0 I ± 2.8
3	[O_3_]: 3.0 gh^−1^	53.5 S ± 2.1	29.5 S ± 4.9
4	Solar O_3_–[O_3_]: 0.9 gh^−1^	33.5 S ± 7.8	8.5 S ± 4.9
5	Solar O_3_–[O_3_]: 3.0 gh^−1^	18.5 I ± 2.1	43.5 I ± 0.7
6	Catalytic O_3_–[O_3_]: 3.0 gh^−1^- [Fe_3_O_4_]: 3.0 gL^−1^*	24.0 I ± 0.0	28.5 I ± 0.7
7	Photocatalytic O_3_–[O_3_]: 1.8 gh^−1^–[TiO_2_]: 50 mgL^−1^	43.0 I ± 4.2	56.5 I ± 2.1
8	O_3_/H_2_O_2_—[O_3_]: 1.8 gh^−1^–[H_2_O_2_]: 50 mgL^−1^	37.5 I ± 2.1	41.0 I ± 1.4

*S* stimulation (increase in *A. fischeri* bioluminescence), *I* inhibition (decrease in *A. fischeri* bioluminescence)

*pH = 9

The toxicity of the treated effluents is expressed as the percentage effect on the bioluminescence of the test microorganism. For accurate data interpretation, values denoted by (I) indicate inhibition, representing a decrease in *A. fischeri* bioluminescence associated with the acute toxicity of the sample. Conversely, values denoted by (S) indicate stimulation, reflecting an increase in *A. fischeri* bioluminescence, meaning favorable conditions for microorganism living.

Ozonation alone exhibited a marked duality: at low doses (0.9 gh^−1^), it induced a strong stimulation of bioluminescence (83% S), suggesting the formation of less harmful byproducts, clearly shown by the high mineralization rates obtained. However, at higher ozone doses (3.0 gh^−1^), with similar removal but in 10 min less time, inhibition progressively increased (up to 53.5% I), indicating that intense oxidation favored the generation of toxic intermediates more persistent than the parent compound. In contrast, the atypical high inhibition observed at an O_3_ dose of 1.8 gh^−1^ can be explained by partial oxidation dynamics. This specific dose likely favors the rapid cleavage of the aromatic ring but leads to a transient accumulation of highly toxic by-products. These intermediates can only be completely oxidized at higher ozone doses or extended exposure times.

In the case of solar ozonation, the quality of intermediate products improved compared to only ozonation. Although mineralization remained modest (6.6–17%), the combination with UV radiation promoted a more selective transformation, resulting in stimulation at an ozone dosage of 0.9 gh^−1^ and low to medium inhibition (after 30 min of exposure), at the maximum dose of 3.0 gh^−1^ following the same trend as when O_3_ alone was applied at higher dosage. This suggests that ultraviolet light enhances bond cleavage and prevents the accumulation of bioactive species, even under moderate ozone doses.

Prolonged catalytic ozonation (Fe_3_O_4_, 3 gL^−1^, 240 min of treatment) achieved a 61% reduction in toxicity compared to untreated technical DQ (28.5% I vs. 55% I), attributable to the extended action of the catalyst, which likely facilitated the final oxidation of toxic intermediates into inert compounds. This result underscores that treatment time may be a decisive parameter for detoxification, even beyond the extent of total mineralization.

Throughout the treatment, it became evident that although the parent compound is almost completely removed, the generated transformation products (TPs) exhibit acute toxicity. In fact, photocatalytic ozonation, despite achieving complete removal and a high mineralization rate (79%), produced the highest recorded inhibition (56.5% I). This value is directly comparable to that obtained for the initial untreated sample, indicating that the highly oxidizing conditions of the system promoted the transient formation of markedly toxic oxidation by-products (OPs). According to the literature, the nature of these by-products depends on the degradation pathway; for PQ, the initial electrophilic attack triggers the opening of the pyridine rings, leading to the formation of aromatic by-products, carboxylic acids, and protonated pyridinium rings (Kumalayanti et al. 2025; Rabaaoui et al. 2025), with monopyridone emerging as a key intermediate (Rabaaoui et al. 2025). Conversely, the degradation of DQ, through processes of demethylation, hydroxylation, and C–C bond cleavage, yields primary intermediates such as monoquat (F. Wang et al. 2025). The transient accumulation of these physicochemical degradation derivatives accounts for the increase in the acute toxicity of the effluent.

Similar behavior was observed when ozonation with hydrogen peroxide was applied, maintaining moderate inhibition (41% I), comparable to that of untreated technical DQ. The addition of H_2_O_2_ enhanced •OH radical generation but was insufficient to eliminate the persistence of toxic metabolites, thereby limiting its effectiveness as a stand-alone strategy.

According to evidence reported in the literature, the reduction of toxicity through ozonation largely depends on process design rather than solely on the elimination of the parent contaminant. A key aspect is that the intermediate products generated during oxidation can determine the final toxicity of the effluent (Carbajo et al. 2015; Du et al. 2020; Völker et al. 2019). Thus, while simple ozonation can effectively remove the target compound, it may also induce the accumulation of more stable and harmful derivatives, a phenomenon that intensifies at higher ozone doses, which is consistent with the results of this study.

Regarding advanced and combined processes, several authors have highlighted that the incorporation of UV radiation, hydrogen peroxide, or catalysts substantially alters the byproduct profile. UV radiation, for instance, has been shown to promote selective bond cleavage, thereby reducing the accumulation of bioactive species. However, photocatalytic configurations have been associated with the generation of secondary compounds of high toxicity, even under conditions of extensive mineralization (Carbajo et al. 2015; Völker et al. 2019). Similarly, the addition of H₂O₂ intensifies hydroxyl radical production, although it does not guarantee complete removal of persistent toxic metabolites on its own (Völker et al. 2019).

Finally, it has been emphasized that detoxification does not depend exclusively on the degree of mineralization achieved, but also on the fate of intermediate products. Several studies have shown that extending the contact time or implementing post-treatments can promote the oxidation of these byproducts into more inert species, thereby significantly reducing residual toxicity (Paździor et al. 2017). This reinforces the notion that operational parameters such as treatment duration may be as decisive as mineralization efficiency itself in mitigating toxicological risks, and that toxicity assays must always be performed to ensure the safety of the treated effluent.

#### *A. franciscana* acute toxicity analysis

The bioassays with *Artemia franciscana* allowed the evaluation of the mortality percentages along the different ozone-based technologies applied to the degradation of technical DQ. It must be highlighted that the control sample showed 100% survival, confirming viability and the absence of cross-contamination. Therefore, it is a similar behavior to that shown for the control sample. There is a big difference if we compare this result with the inhibition percentages obtained with *A. fischeri* for the initial 10 mgL^−1^ of technical DQ, which reveals the necessity of performing at least two bioassays to accurately evaluate ecotoxicity in different organisms that could show different sensitivities. Ozonation alone showed, again, a dual behavior: at low ozone dose (0.9 gh^−1^), it resulted in almost a complete survival of *Artemia*, though, at higher doses (3.0 gh^−1^), with identical removal and similar mineralization, 17% mortality was recorded, indicating that intense oxidation promoted the formation of stable toxic intermediates.

In contrast, solar ozonation exhibited greater predictability: at low ozone dosage, in combination with UV light, no mortality was detected, confirming that radiation promotes selective degradation pathways leading to innocuous products. At higher ozone doses, mortality of 20% was observed, confirming that stronger oxidant conditions provoke the generation of slightly more toxic metabolites. Conversely, photocatalytic ozonation, despite achieving complete removal and 79% mineralization, produced 67% mortality in *Artemia,* which is also in concordance with the results obtained with *A. fischeri*. In addition, prolonged catalytic ozonation (Fe_3_O_4_ 3 gL^−1^, 240 min) showed even higher mortality (80%) than the TiO_2_ system, demonstrating the higher sensitivity of *Artemia* to this catalyst.

Ozonation combined with hydrogen peroxide also generated 100% mortality, showing that peroxide addition did not improve product quality but rather intensified the formation of lethal bioactive species.

Thus, while simple and solar ozonation can achieve effective detoxification, photocatalytic and catalytic ozonation pose hidden risks that compromise the effluent’s environmental viability and reuse.

The literature supports some of these observations. It has been reported that in *Artemia franciscana* bioassays, no relevant toxic effects were detected after conventional ozonation, suggesting that the process can be safe under certain operational conditions (Poelmans et al. 2023). Specifically, during the pilot-scale treatment of complex reverse-osmosis concentrate (ROC) at an ozone dosage of 100 g O_3_m^−3^, toxicity toward A. franciscana remained negligible even after up to 3 h of continuous ozonation. However, it has also been observed that short ozonation treatments tend to reduce initial toxicity, whereas prolonged exposures may lead to the accumulation of ecotoxic byproducts. This accumulation was clearly evidenced by the high sensitivity of other marine organisms, such as the algae *Phaeodactylum tricornutum*, which showed increased growth inhibition after extended ozonation. Nevertheless, A. franciscana has shown relatively low sensitivity to such oxidative byproducts, which explains why significant mortality is not always evident. In fact, its low sensitivity is comparable to that of *Daphnia magna*, with studies indicating that any minor toxic effects observed in these organisms are primarily attributed to the high conductivity and salinity of the wastewater matrix rather than the generated oxidation intermediates (Poelmans et al. 2023).

A critical aspect is the absence of studies directly analyzing ozonized pesticide residues in *A. franciscana*. Although this species is recognized as a bioindicator for pesticide toxicity, most research has focused either on direct pesticide exposure without prior treatment or on other contaminants, without addressing how ozonation modifies biological effects (Albarano et al. 2022; Favero et al. 2025).

In this regard, available studies indicate that PQ is highly toxic to both larvae and adults of *A. franciscana*, causing significant adverse effects across different life stages (Rahnama et al. 2018). Although the DQ effect has not been directly assessed on this species, its chemical similarity and shared mechanism of reactive oxygen species generation suggest a comparable toxicity profile. This reinforces the importance of considering not only contaminant removal and mineralization but also the nature of the byproducts generated during ozonation, as pesticides of this class and their oxidized derivatives may retain or even enhance ecotoxicological risks.

## Conclusions

Solar photolysis and the use of magnetite (Fe_3_O_4_) as a catalyst did not promote significant degradation or mineralization of PQ and DQ under any evaluated condition, evidencing that these herbicides are highly resistant and that more oxidative processes are required for their removal.

Simple ozonation showed higher selectivity toward DQ compared to PQ, with limited mineralization. Increasing the ozone dose improved mineralization up to 50%, but at the expense of higher energy consumption. From the results obtained in this work, 1.8 gh^−1^ of O_3_ has been selected at the best dosage, balancing efficiency and sustainability.

The introduction of magnetite reduced degradation and mineralization efficiency compared to simple ozonation. Although high catalyst concentrations and basic pH accelerated the initial degradation of DQ, final mineralization remained low. These results suggest that the catalyst interferes with ozone-derived radicals or favors the formation of stable byproducts, limiting complete contaminant conversion.

The combination of ozone, solar radiation, and TiO_2_ (50 mgL^−1^) significantly improved degradation and mineralization compared to solar ozonation and catalytic ozonation, evidencing the synergy between sunlight and ozone for the generation of hydroxyl radicals and the conversion of recalcitrant byproducts into more biocompatible compounds.

This study demonstrates that although simple ozonation is effective for the partial degradation of PQ and DQ, photocatalytic ozonation represents the most promising and sustainable alternative for the removal of bipyridyl herbicides in aqueous matrices, and states that the contribution to mineralization by the combined process was substantially higher (79% vs. 6.78%, respectively). Photocatalytic ozonation optimizes the generation of reactive oxygen species and reduces the formation of persistent byproducts.

Toxicity assays with *A. fischeri* and *A. franciscana* confirmed that degradation and mineralization alone are insufficient to assess process effectiveness and safety for treated effluent reuse. While simple ozonation reduced toxicity at moderate doses, incomplete mineralization or stronger oxidant conditions allowed harmful intermediates to persist. Solar-assisted ozonation showed the best balance, as UV radiation promoted selective pathways leading to detoxification. In contrast, photocatalytic ozonation with TiO_2_, despite achieving complete pesticide removal, generated the highest final toxicity due to the presence of highly stable toxic byproducts.

## Data Availability

Data will be available on reasonable request.
